# Supplementary material for: Framework engineering to produce dominant T cell receptors with enhanced antigen-specific function
Source: Nat Commun. 2019 Oct 1;10:4451. doi: 10.1038/s41467-019-12441-w (PMC6773850; doi:10.1038/s41467-019-12441-w)
Supplement: Supplementary file 1 — Supplementary Information [file 41467_2019_12441_MOESM1_ESM.pdf]

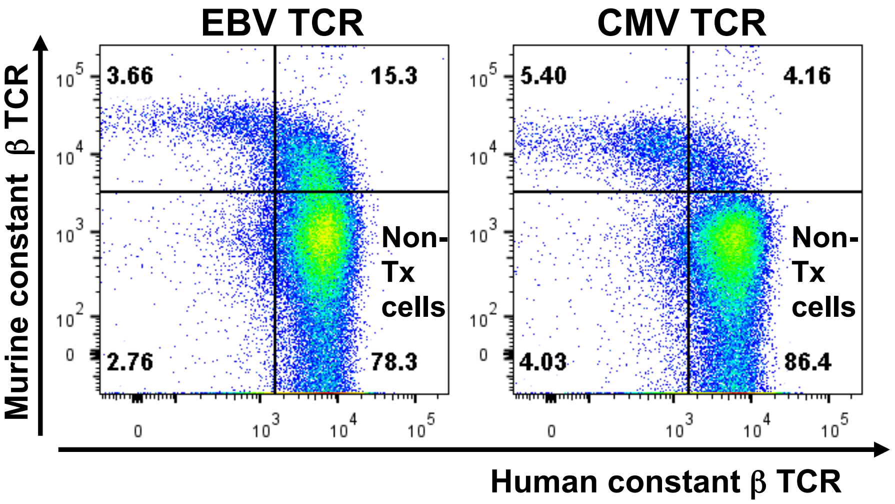


**Supplementary Figure 1: Identification of dominant and weak human TCRs.** Dot plots of polyclonal human peripheral blood T cells transduced with synthetic dominant TCRs. Left panel: EBV TCR (specific for the LMP2 peptide). Right panel: CMV TCR (specific for the pp65 peptide). Transduced cells were double-stained with anti-human constant region antibodies to identify the endogenous TCR and anti-murine constant region antibodies to identify the introduced TCR. Live, single cells were first gated on CD3 and CD8. Non-transduced (non-Tx) T cells are labelled.

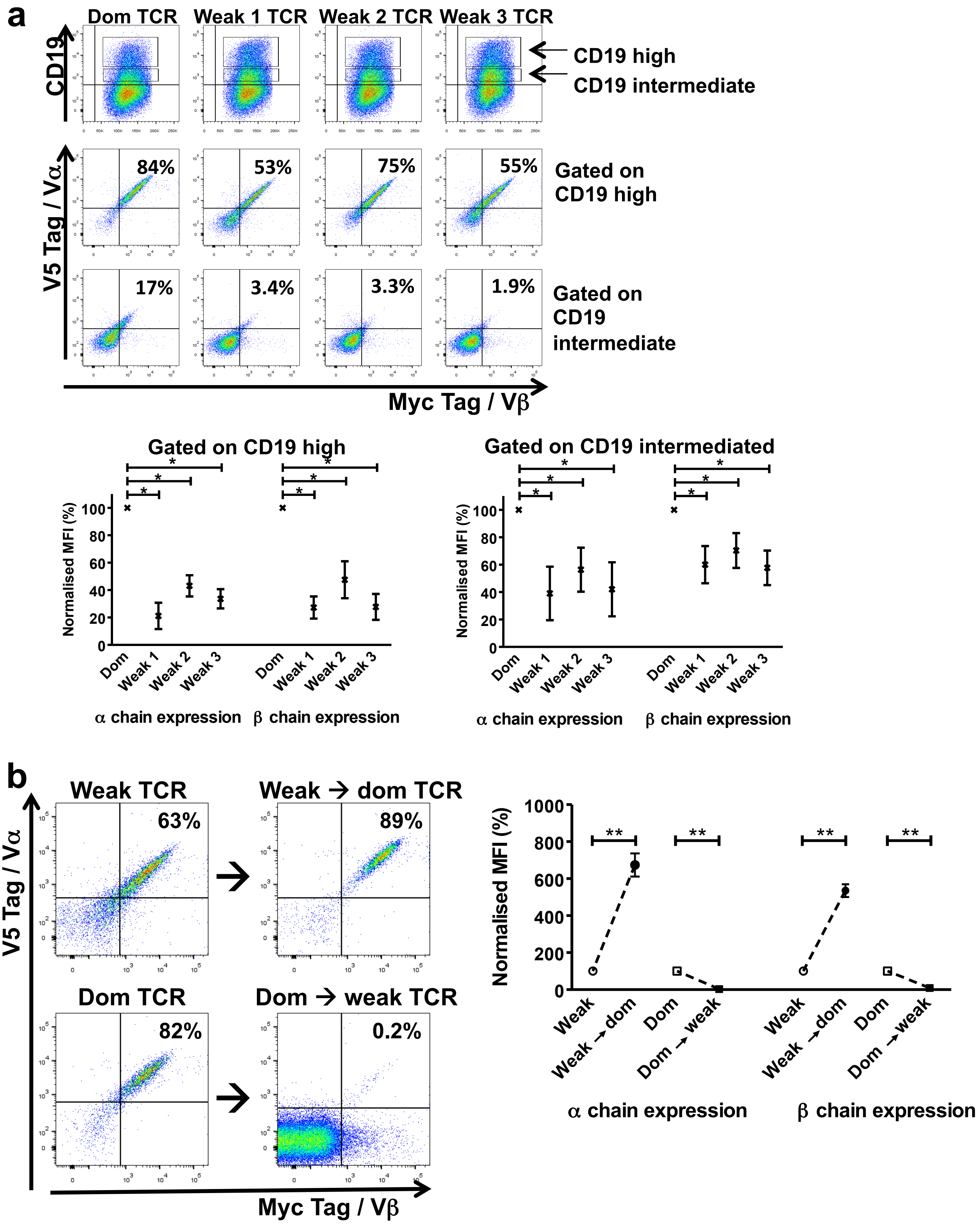


**Supplementary Figure 2: Conversion of a weak TCR into a dominant TCR by replacement of 14 variable region framework residues.** (a) Representative example of n=4 independent experiments showing human Jurkat cells (lacking an endogenous TCR) transduced with a dominant (Dom) TCR (TRAV38-2/TRBV7-8) or 3

weak TCRs: weak 1(TRAV13-2/TRBV7-3), weak 2 (TRAV23/TRBV7-9) or weak 3 (TRAV29/TRBV2). Top panel: CD19 expression levels. Middle panel: TCR $\alpha$ and $\beta$ chain expression levels on gated CD19$^{high}$ cells. Bottom panel: TCR $\alpha$ and $\beta$ chain expression levels on gated CD19$^{intermediate}$ cells. V$\alpha$, variable alpha. V$\beta$, variable beta. Right panels: pooled data (means ± SEM) showing TCR $\alpha$ and $\beta$ chain expression levels on CD19$^{high}$ cells (top) and CD19$^{intermediate}$ cells (bottom) normalized to the Dom TCR. n=4 independent experiments. * $P < 0.05$ (Mann-Whitney U test) for all comparisons between the Dom TCR $\alpha$ chain and the weak TCR $\alpha$ chains and for all comparisons between the Dom TCR $\beta$ chain and the weak TCR $\beta$ chains. MFI, median fluorescence intensity. (b) Left top panel: introduction of the 14 residues indicated in Figure 1e into the weak 1 TCR (TRAV13-2/TRBV7-3) generated the weak → dom TCR with enhanced $\alpha/\beta$ expression on Jurkat cells lacking an endogenous TCR. Left bottom panel: replacement of the 14 residues in the Dom TCR (TRAV38-2/TRBV7-8) with the equivalent residues in the weak 1 TCR (TRAV13-2/TRBV7-3) generated the dom → weak TCR with undetectable $\alpha/\beta$ expression on Jurkat cells lacking an endogenous TCR. Data are representative of n=5 independent experiments. Right panel: pooled data (means ± SEM ) showing TCR $\alpha$ and $\beta$ chain expression levels normalized to the corresponding unmodified TCRs. n=5 independent experiments. ** $P < 0.001$ (Mann-Whitney test) for all comparisons between the modified TCRs and the corresponding unmodified TCRs. MFI, median fluorescence intensity.

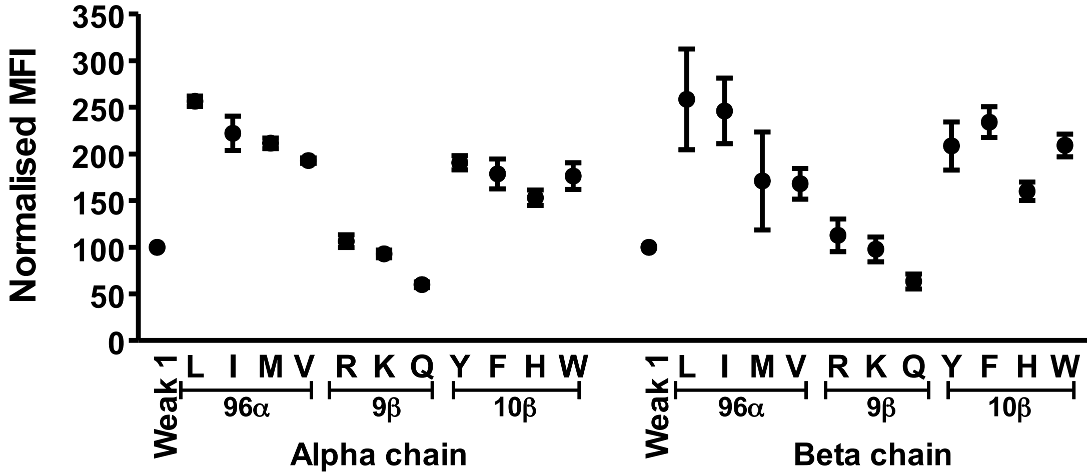


**Supplementary Figure 3: Biochemically similar amino acids at positions 96α, 9β, 10β have similar effects on TCR expression.** Jurkat cells (expressing an endogenous TCR) were transduced with the weak 1 wild-type TCR or the modified versions containing either leucine, isoleucine, methionine, valine at position 96α, or arginine, lysine, glutamine at 9β, or tyrosine, phenylalanine, tryptophane, histidine at 10β. TCR α/β surface expression was determined in gated cells expressing equivalent levels of CD19. Pooled data (means ± SEM ) normalized to the weak 1 unmodified TCR. n=3 independent experiments. MFI, median fluorescence intensity.

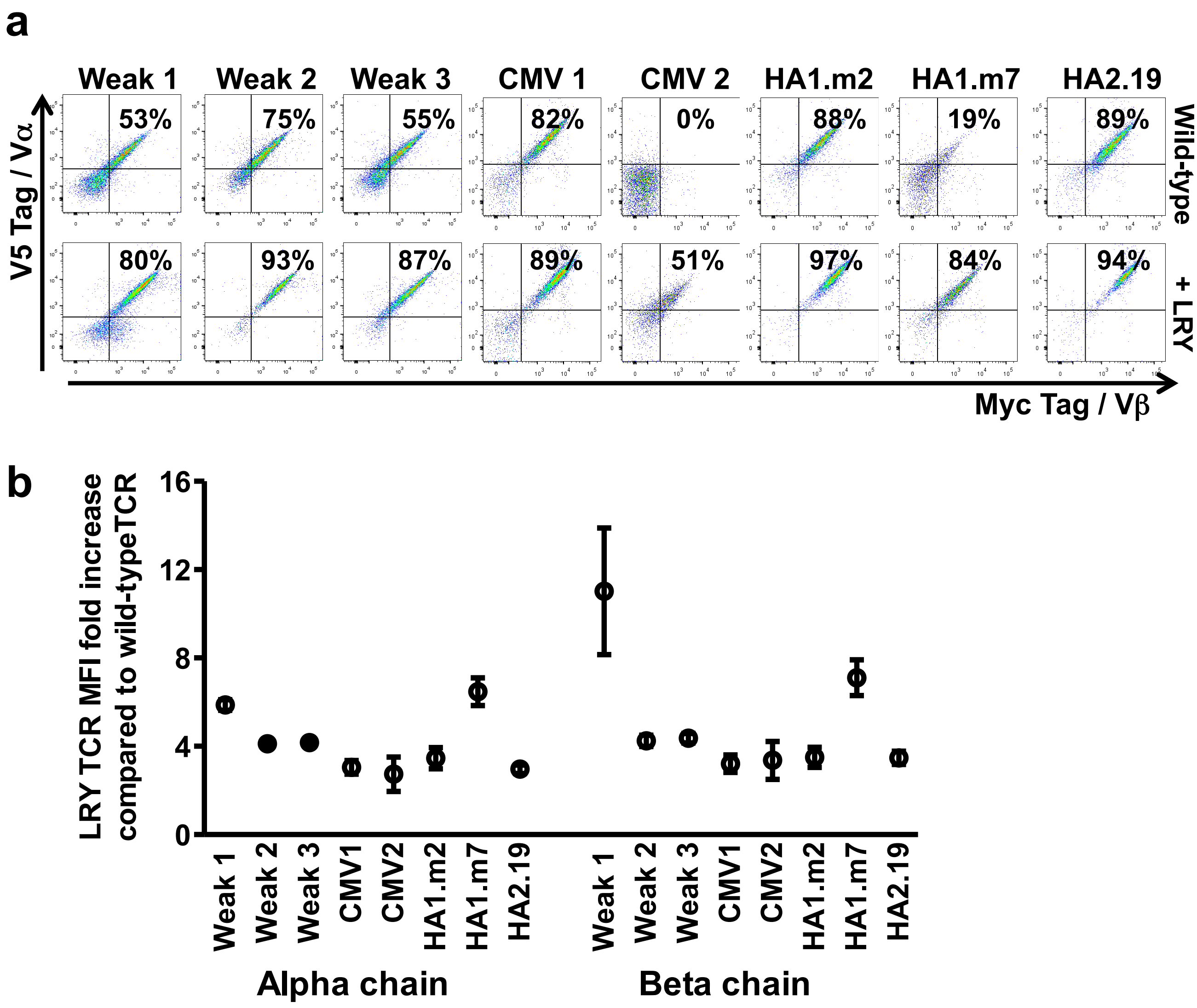


**Supplementary Figure 4: Replacement of three framework residues consistently enhances TCR expression.** The roles of L96α, R9β and Y10β (LRY) were tested in 3 weak TCRs selected from the weak TCR library and in 5 antigen-specific TCRs (2 TCRs specific for CMVpp65, 2 TCRs specific for HA1, and 1 TCR specific for HA2). (a) A representative example of n=3 independent experiments showing Jurkat cells (lacking an endogenous TCR) transduced with the indicated wild-type TCRs (top row) or the corresponding LRY-modified TCRs containing L96α, R9β and Y10β (bottom row). TCR α/β surface expression was determined on gated cells expressing equivalent levels of CD19. (b) Pooled data (means ± SEM) showing the fold increase in TCR α and β chain expression for 8 LRY-modified TCRs compared with the corresponding wild-type TCRs. n=3 independent experiments. MFI, median fluorescence intensity.

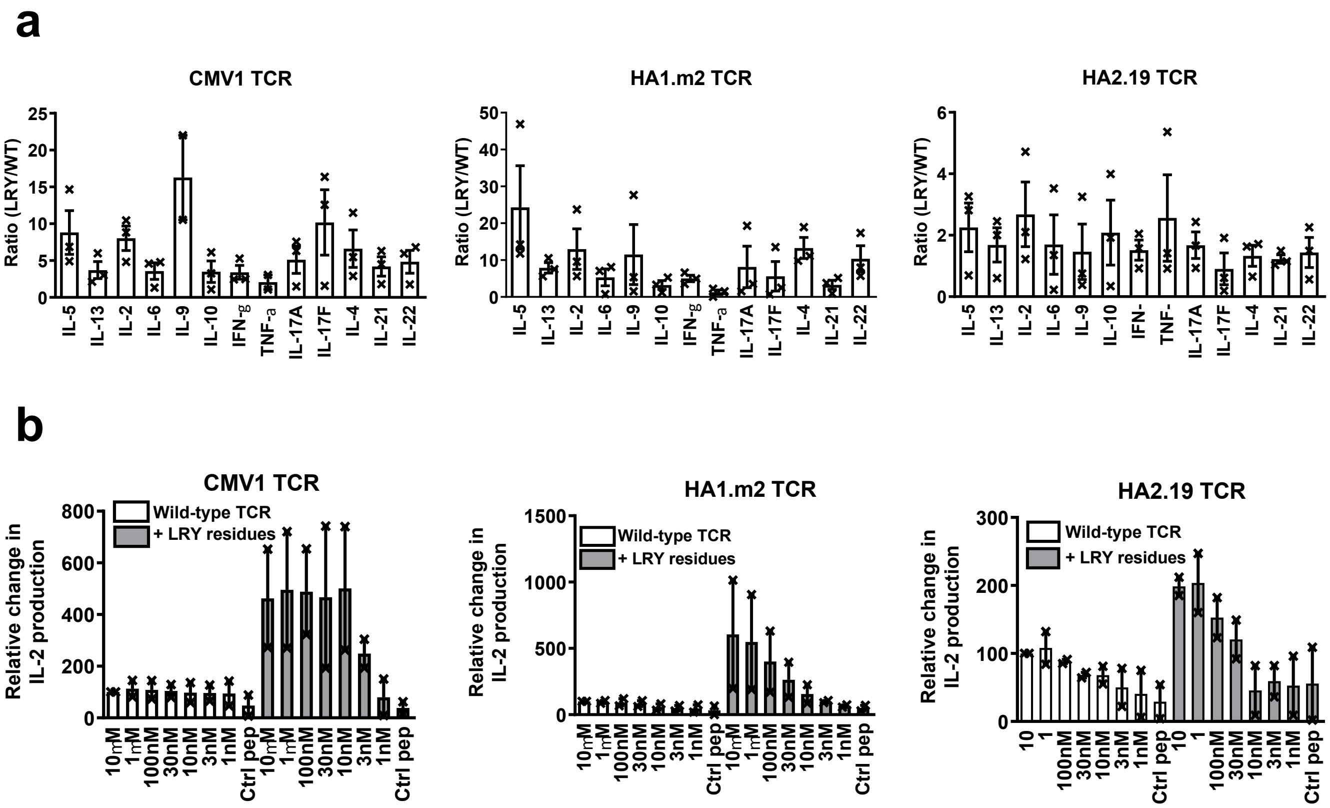


**Supplementary Figure 5: Replacement of three framework residues increases cytokine production and peptide sensitivity.** (a) Transduced T cells were stimulated overnight with cognate peptide-loaded T2 cells. Supernatants were harvested and tested for secreted cytokines using a LEGENDplex Human Th Cytokine Panel. Data were pooled (means ± SEM) and expressed as the ratio of cytokine production by T cells expressing L96$\alpha$, R9$\beta$ and Y10$\beta$ (LRY)-modified TCRs- over cytokine production by T cells expressing wild-type TCRs. n=3 independent experiments. (b) Transduced T cells were stimulated overnight with T2 cells loaded with the indicated concentrations of cognate peptide. IL-2 production was measured by ELISA. Open bars: T cells expressing wild-type TCRs. Grey bars: T cells expressing LRY-modified TCRs. Data were pooled (means ± SEM) and normalized to IL-2 production by wild-type TCR-transduced T cells stimulated with T2 cells loaded with cognate peptide at 10 $\mu$M. n=2 independent experiments.

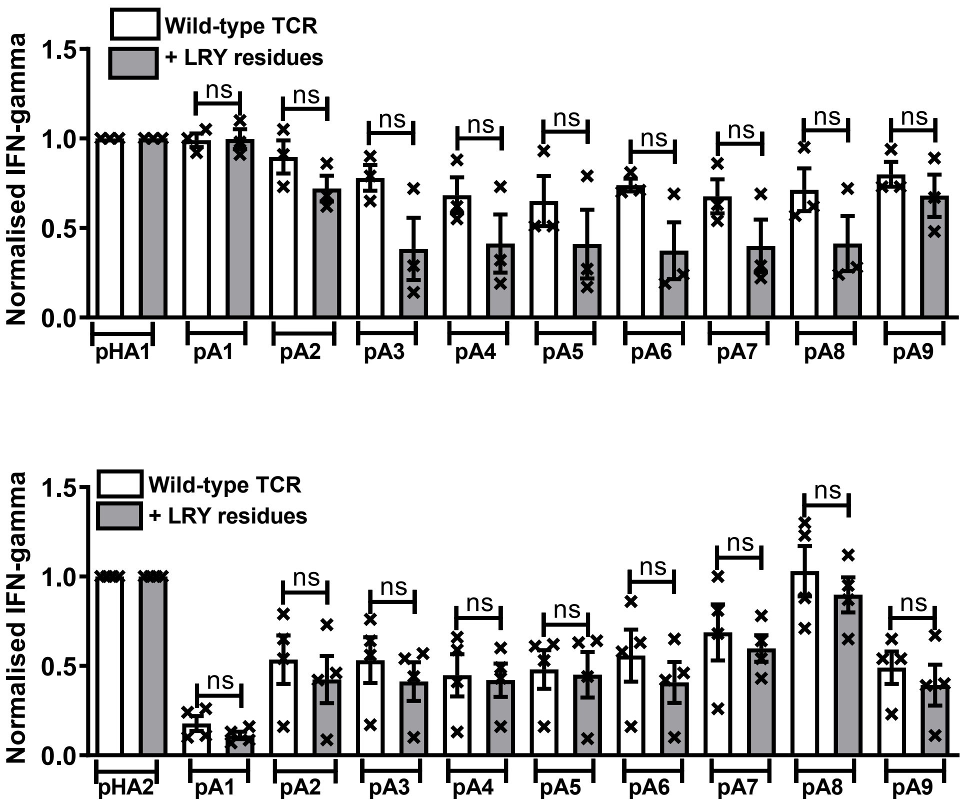


**Supplementary Figure 6: Replacement of three framework residues does not affect peptide specificity.** Transduced T cells with similar transduction efficiencies were stimulated overnight with variant peptide-loaded or cognate peptide-loaded T2 cells. IFNγ production was measured by ELISA. Data are shown pooled and normalized to the amount of IFNγ produced in response to stimulation with the cognate peptide. Top panel: T cells expressing the wild-type (open bars) or the LRY-modified (grey bars) HA1.m2 TCR. Data were pooled (means ± SEM) from n=3 independent experiments. Bottom panel: T cells expressing the wild-type (open bars) or the LRY-modified (grey bars) HA2.19 TCR. Data were pooled (means ± SEM) from n=4 independent experiments. ns (non-significant), $P > 0.05$ (Mann-Whitney U test) for all comparisons between the LRY-modified TCRs and the corresponding wild-type TCRs.

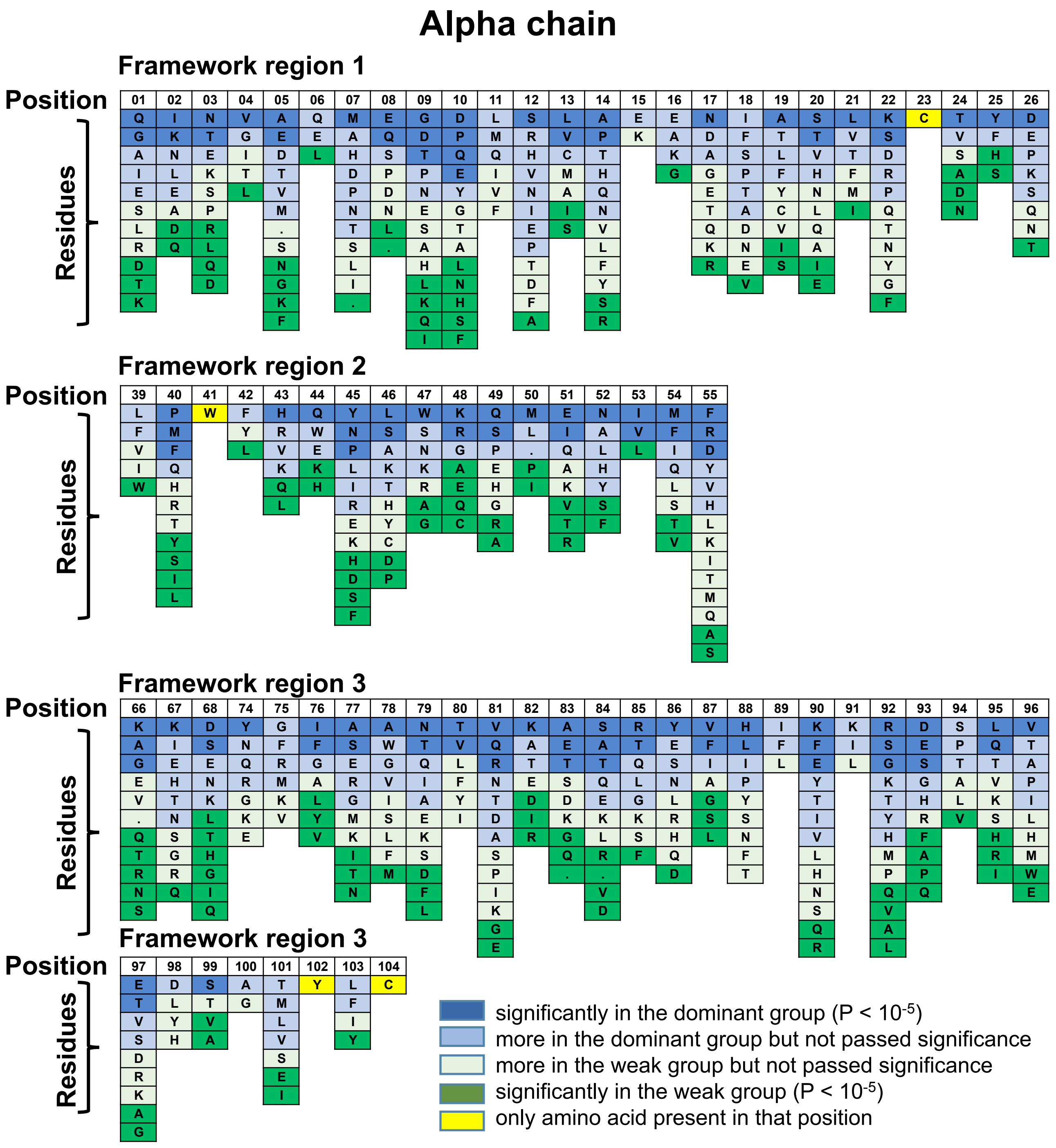


**Supplementary Figure 7: Amino acid frequencies in dominant and weak alpha chains.** Transduced T cells expressing weak endogenous TCRs or dominant endogenous TCRs were sort-purified by flow cytometry. The endogenous TCRs were then sequenced using next generation sequencing to generate 53,309 distinct alpha clonotypes. Cochran–Mantel–Haenszel test was used to compare the amino acid frequency at every position of the IMGT variable framework regions FR1, FR2 and FR3 between the dominant and weak alpha TCRs. Shown are residues that passed the multiple comparisons ($P<10^{-5}$) and are significantly more frequent in the dominant group (dark blue) or the weak group (dark green). Also shown are residues that were more frequent, but did not pass significance ($0.05 < P < 10^{-5}$) in the dominant group (light blue) or the weak group (light green). Residues in yellow are the only amino acid present at that position.

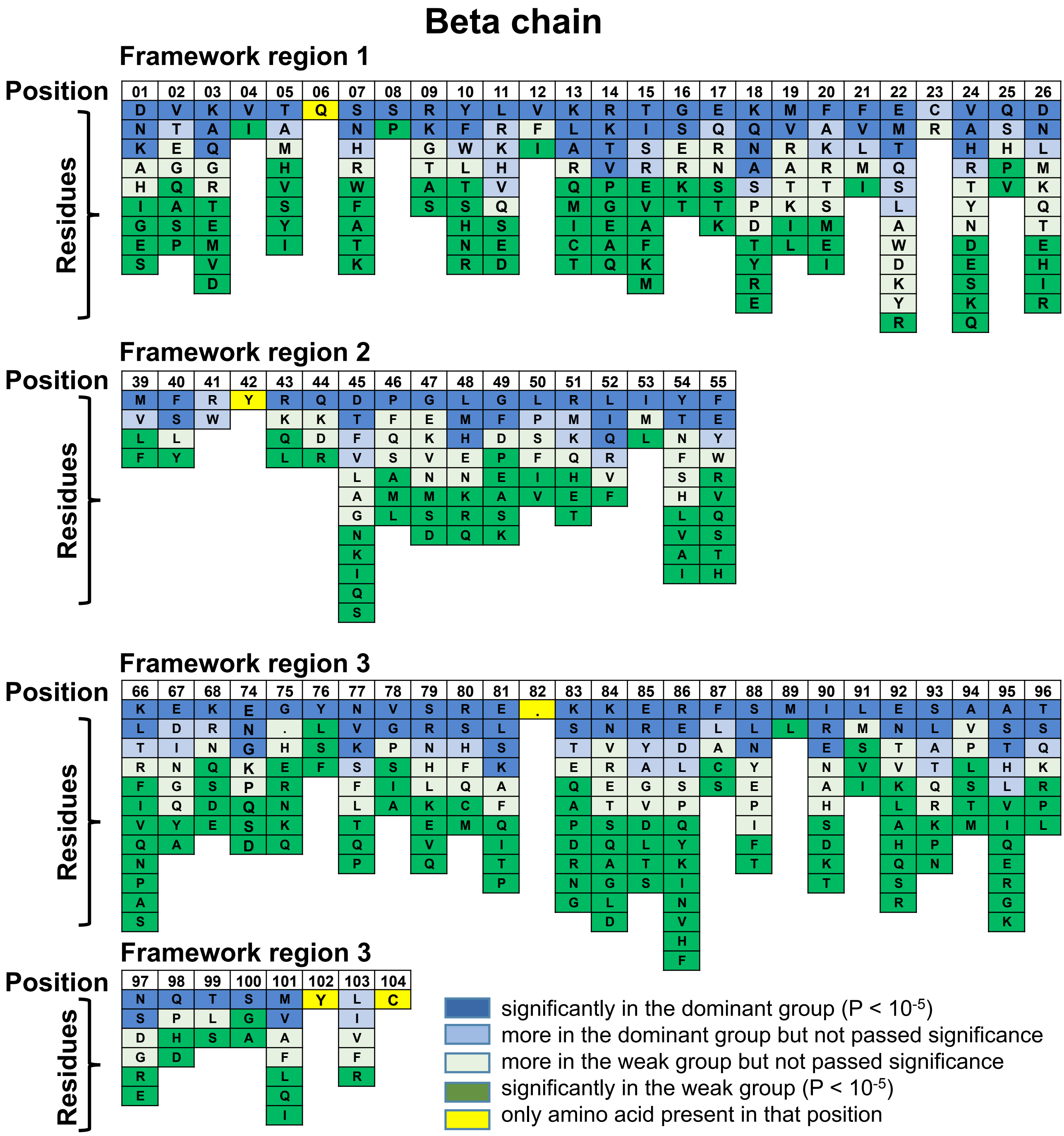


**Supplementary Figure 8: Amino acid frequencies in dominant and weak beta chains.** Transduced T cells expressing weak endogenous TCRs or dominant endogenous TCRs were sort-purified by flow cytometry. The endogenous TCRs were then sequenced using next generation sequencing to generate 80,921 distinct beta clonotypes. Cochran–Mantel–Haenszel test was used to compare the amino acid frequency at every position of the IMGT variable framework regions FR1, FR2 and FR3 between the dominant and weak beta TCRs. Shown are residues that passed the multiple comparisons ($P<10^{-5}$) and are significantly more frequent in the dominant group (dark blue) or the weak group (dark green). Also shown are residues that were more frequent, but did not pass significance ($0.05 < P < 10^{-5}$) in the dominant group (light blue) or the weak group (light green). Residues in yellow are the only amino acid present at that position.

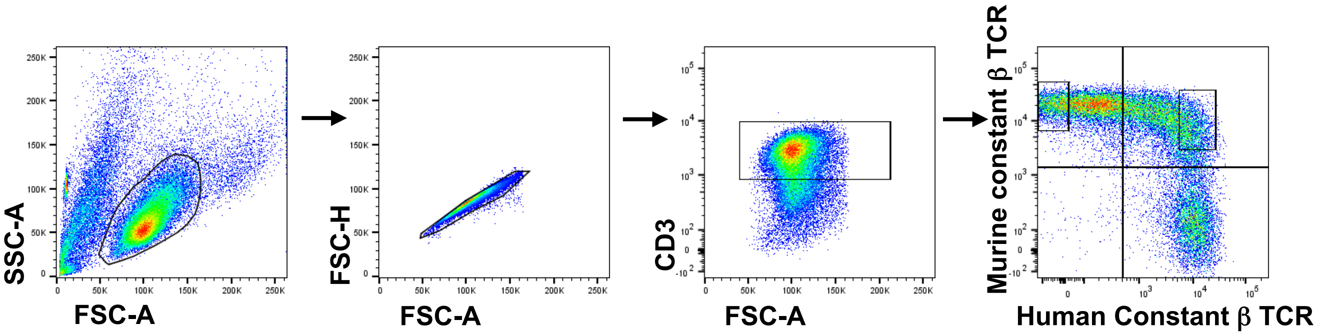


**Supplementary Figure 9: Gating strategy used for cell sorting.** Shown is the gating strategy used for cell sorting of CD3+ T cells that express endogenous dominant and weak TCRs. This strategy was used in Figures 1c, 1d, 1e and Supplementary Figures 7 and 8 and Supplementary Tables 1 and 2.

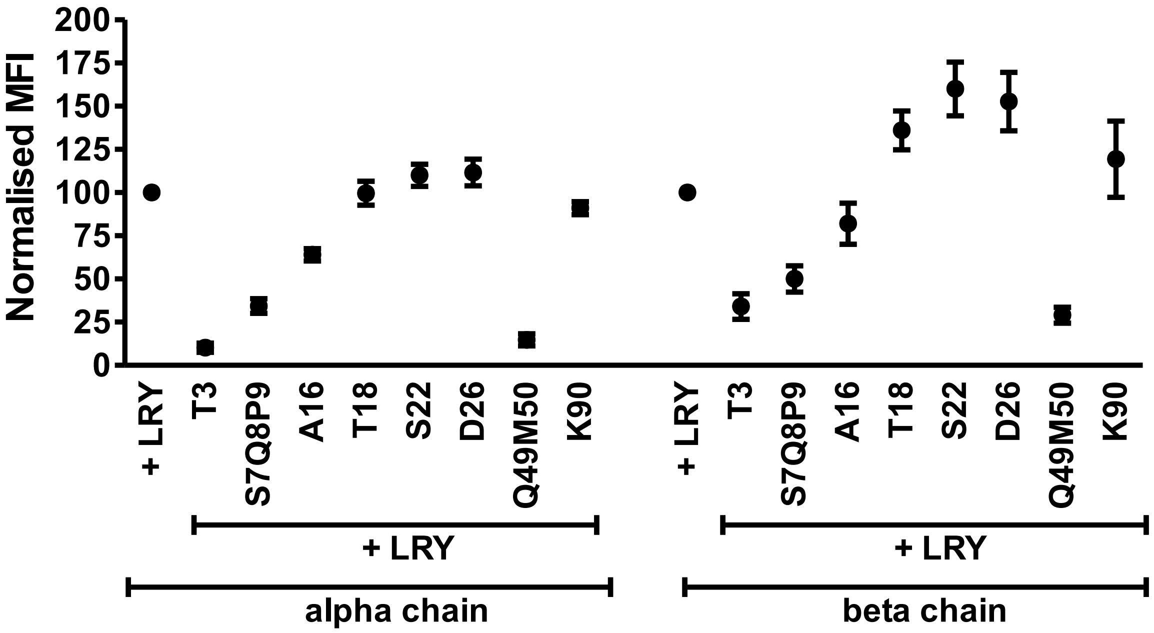


**Supplementary Figure 10: The alpha chain residues T18, S22, D26 mediate a modest improvement in expression of LRY-modified TCRs**. The LRY-version of the human CMV1 TCR was further modified to introduce the alpha chain residues T3, S7+Q8+P9, A16, T18, S22, D26, Q49+M50, K90. These residues were selected because they were present in the dominant alpha chains V$\alpha$38-1, V$\alpha$38-2 and V$\alpha$9-2 and rare in the remaining 44 alpha chains of the IMGT database. Jurkat cells were transduced with the LRY-TCR or variants containing the additional residue modifications. TCR expression was assessed in gated CD19-positive Jurkat cells. Data were pooled (means ± SEM) and shown is the MFI (mean fluorescent intensity) of all variants relative to the expression levels of the LRY-TCR. n=3 independent experiments.

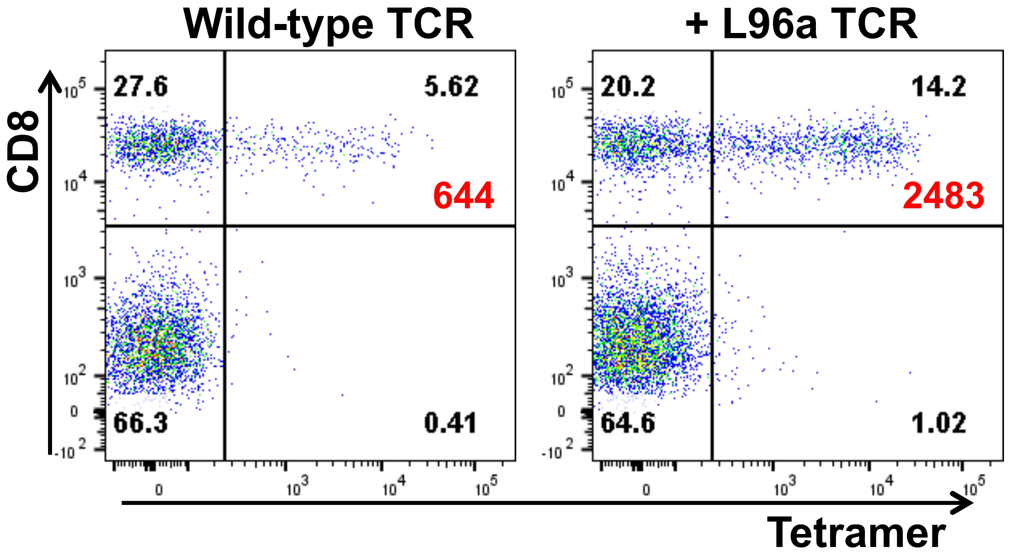


**Supplementary Figure 11: The introduction of leucine at position 96 of the alpha chain is sufficient to improve TCR expression.** A TCR already containing a native R9β/Y10β, but lacking leucine at position 96α was engineered to introduce L96α. Human peripheral blood T cells were transduced with the wild-type TCR and the L96α-modified variant. Shown is the percentage of gated CD19-positive CD8 T cells that bound tetramer. Black numbers indicate the percentage of T cells in each quadrant and red numbers indicate the tetramer MFI (mean fluorescent intensity) of gated CD19-positive CD8 T cells. The figure is representative of at least n=6 independent experiments with similar results.

# Alpha chain

**FR1**

| Position | 01 | 02 | 03 | 04 | 05 | 06 | 07 | 08 | 09 | 10 | 11 | 12 | 13 | 14 | 15 | 16 | 17 | 18 | 19 | 20 | 21 | 22 | 23 | 24 | 25 | 26 |
|---|---|---|---|---|---|---|---|---|---|---|---|---|---|---|---|---|---|---|---|---|---|---|---|---|---|---|
| Library 1 | | | T | V | T | | S | Q | P | E | M | | | | | A | E | T | V | T | L | S | | T | | D |
| Library 2 | Q | I | T/N | V | A | | M | Q/E | G | E/D | | S | L | A | | A/E | N | | A | T/A | L | S/K | | T | Y | D |

**FR2**

| Position | 39 | 40 | 41 | 42 | 43 | 44 | 45 | 46 | 47 | 48 | 49 | 50 | 51 | 52 | 53 | 54 | 55 |
|---|---|---|---|---|---|---|---|---|---|---|---|---|---|---|---|---|---|
| Library 1 | L | P | | | | | P | P | S | R | Q | M | I | L | V | | R |
| Library 2 | | P/F | | | H | Q | P/Y | L | S/W | R/K | Q | M | I/E | N | V/I | M | R/F |

**FR3**

| Position | 66 | 67 | 68 | 74 | 75 | 76 | 77 | 78 | 79 | 80 | 81 | 82 | 83 | 84 | 85 | 86 | 87 | 88 | 89 | 90 | 91 | 92 | 93 | 94 | 95 | 96 |
|---|---|---|---|---|---|---|---|---|---|---|---|---|---|---|---|---|---|---|---|---|---|---|---|---|---|---|
| Library 1 | A | T | E | N | | F | S | V | N | F | Q | K | A | A | K | S | F | | | K | | S | D | S | | L |
| Library 2 | A/K | K | E/D | Y | | F/I | S | A | N | T | Q/V | K | A/E | A | R | Y | F/V | H | | K | | S/R | D | S | L | V |

| Position | 97 | 98 | 99 | 100 | 101 | 102 | 103 | 104 |
|---|---|---|---|---|---|---|---|---|
| Library 1 | G | D | A | | M | | | |
| Library 2 | E | D | S | | T | | L | |

# Beta chain

**FR1**

| Position | 01 | 02 | 03 | 04 | 05 | 06 | 07 | 08 | 09 | 10 | 11 | 12 | 13 | 14 | 15 | 16 | 17 | 18 | 19 | 20 | 21 | 22 | 23 | 24 | 25 | 26 |
|---|---|---|---|---|---|---|---|---|---|---|---|---|---|---|---|---|---|---|---|---|---|---|---|---|---|---|
| Library 2 | D | V | K | V | T | | S | | R | Y | L | V | K | K | T | G | | K | M | A | F | E | | V | Q | D |

**FR2**

| Position | 39 | 40 | 41 | 42 | 43 | 44 | 45 | 46 | 47 | 48 | 49 | 50 | 51 | 52 | 53 | 54 | 55 |
|---|---|---|---|---|---|---|---|---|---|---|---|---|---|---|---|---|---|
| Library 2 | M | F | | | R | Q | T | P | G | L | G | L | R | L | I | Y | F |

**FR3**

| Position | 66 | 67 | 68 | 74 | 75 | 76 | 77 | 78 | 79 | 80 | 81 | 82 | 83 | 84 | 85 | 86 | 87 | 88 | 89 | 90 | 91 | 92 | 93 | 94 | 95 | 96 |
|---|---|---|---|---|---|---|---|---|---|---|---|---|---|---|---|---|---|---|---|---|---|---|---|---|---|---|
| Library 2 | L | E | K | E | G | Y | N | V | S | R | E | | K | N | E | R | F | S | M | I | L | N | S | A | A | T |

| Position | 97 | 98 | 99 | 100 | 101 | 102 | 103 | 104 |
|---|---|---|---|---|---|---|---|---|
| Library 2 | N | Q | T | S | M | | L | |

**Supplementary Table 1: Significantly increased frequencies of particular amino acids at certain positions in dominant TCRs.** Transduced T cells expressing weak endogenous TCRs or dominant endogenous TCRs were sort-purified by flow cytometry. The endogenous TCRs were then sequenced using Sanger sequencing to generate library 1 (884 distinct clonotypes), or next generation sequencing to generate library 2 (130,000 distinct clonotypes). Fisher's exact test was used to compare the frequency of specific amino acids at every position of the IMGT variable framework regions FR1, FR2 and FR3. Supplementary Table 1a: The TCR$\alpha$ analysis of library 1 was performed without correction for multiple comparisons and the amino acids at each position that were mostenriched in the dominant TCR library are listed ($p<0.05$). The TCRa analysis of library 2 was performed with correction for multiple comparisons using the Bonferroni correction and amino acids that were most enriched in the dominant TCR library are listed

(p<0.00001). More than 1 amino acid was enriched in many positions. Indicated are the amino acids that were enriched in both libraries 1 and 2, and also amino acids that were most significantly enriched in library 2. Supplementary Table 1b: The TCRb analysis was performed on library 2 only. The analysis was corrected for multiple comparisons using the Bonferroni correction. The most significantly enriched amino acids in the dominant TCR library are listed (p<0.00001).

**a** **Variable alpha**

| | Dominant (%) | Weak (%) |
|---|---|---|
| **TRAV1-1** | 0.63 | 1.22 |
| **TRAV1-2** | 1.81 | 2.34 |
| **TRAV2** | 3.52 | 4.96 |
| **TRAV3** | 2.26 | 1.22 |
| **TRAV4** | 2.31 | 2.94 |
| **TRAV5** | 0.97 | 1.88 |
| **TRAV6** | 1.70 | 1.92 |
| **TRAV8-1** | 0.82 | 1.03 |
| **TRAV8-2** | 3.68 | 3.65 |
| **TRAV8-3** | 1.67 | 2.26 |
| **TRAV8-6** | 2.57 | 2.31 |
| **TRAV9-1** | 0.01 | 0.00 |
| **TRAV9-2** | 7.82 | 6.32 |
| **TRAV10** | 1.46 | 1.71 |
| **TRAV12-1** | 2.57 | 3.66 |
| **TRAV12-2** | 4.93 | 2.89 |
| **TRAV12-3** | 2.87 | 2.14 |
| **TRAV13-1** | 8.01 | 5.75 |
| **TRAV13-2** | 1.90 | 2.93 |
| **TRAV14/DV4** | 2.00 | 2.11 |
| **TRAV16** | 1.00 | 1.38 |
| **TRAV17** | 3.38 | 2.78 |
| **TRAV19** | 4.11 | 3.43 |
| **TRAV20** | 2.00 | 1.50 |
| **TRAV21** | 5.86 | 6.12 |
| **TRAV22** | 2.72 | 0.95 |
| **TRAV23/DV6** | 1.95 | 1.86 |
| **TRAV24** | 2.14 | 2.16 |
| **TRAV25** | 1.49 | 1.71 |
| **TRAV26-1** | 1.82 | 2.02 |
| **TRAV26-2** | 2.78 | 3.05 |
| **TRAV27** | 1.76 | 2.22 |
| **TRAV28** | 0.01 | 0.00 |
| **TRAV29/DV5** | 3.50 | 5.65 |
| **TRAV30** | 0.75 | 1.19 |
| **TRAV31** | 0.01 | 0.00 |
| **TRAV34** | 0.33 | 0.53 |
| **TRAV35** | 1.89 | 2.75 |
| **TRAV36/DV7** | 1.82 | 1.58 |
| **TRAV38-1** | 1.72 | 1.30 |
| **TRAV38-2/DV8** | 2.88 | 1.46 |
| **TRAV39** | 1.00 | 1.04 |
| **TRAV40** | 0.34 | 0.25 |
| **TRAV41** | 0.57 | 1.22 |

**b** **Variable beta**

| | Dominant (%) | Weak (%) |
|---|---|---|
| **TRBV1** | 0.02 | 0.06 |
| **TRBV2** | 2.90 | 4.87 |
| **TRBV3-1** | 3.08 | 2.13 |
| **TRBV4-1** | 2.62 | 2.51 |
| **TRBV4-2** | 1.49 | 1.74 |
| **TRBV4-3** | 1.71 | 2.55 |
| **TRBV5-1** | 6.69 | 4.04 |
| **TRBV5-2** | 0.00 | 0.01 |
| **TRBV5-3** | 0.01 | 0.02 |
| **TRBV5-4** | 1.11 | 0.90 |
| **TRBV5-5** | 1.44 | 0.99 |
| **TRBV5-6** | 1.18 | 1.31 |
| **TRBV5-8** | 0.22 | 0.33 |
| **TRBV6-1** | 5.23 | 4.03 |
| **TRBV6-4** | 0.53 | 0.97 |
| **TRBV6-5** | 4.53 | 4.92 |
| **TRBV6-6** | 2.24 | 1.62 |
| **TRBV6-7** | 0.03 | 0.01 |
| **TRBV6-8** | 0.02 | 0.08 |
| **TRBV6-9** | 0.40 | 0.35 |
| **TRBV7-2** | 4.81 | 5.38 |
| **TRBV7-3** | 2.16 | 3.80 |
| **TRBV7-4** | 0.12 | 0.16 |
| **TRBV7-5** | 0.02 | 0.06 |
| **TRBV7-6** | 1.27 | 1.94 |
| **TRBV7-7** | 0.17 | 0.47 |
| **TRBV7-8** | 2.38 | 1.30 |
| **TRBV7-9** | 5.25 | 3.36 |
| **TRBV9** | 3.33 | 5.33 |
| **TRBV10-1** | 0.58 | 0.60 |
| **TRBV10-2** | 0.89 | 0.50 |
| **TRBV10-3** | 3.51 | 2.09 |
| **TRBV11-1** | 0.75 | 0.47 |
| **TRBV11-2** | 3.26 | 3.33 |
| **TRBV11-3** | 0.41 | 0.96 |
| **TRBV12-1** | 0.02 | 0.02 |
| **TRBV12-2** | 0.21 | 0.28 |
| **TRBV12-4** | 2.99 | 4.08 |
| **TRBV12-5** | 0.28 | 0.54 |
| **TRBV13** | 0.73 | 0.87 |
| **TRBV14** | 0.30 | 0.54 |
| **TRBV15** | 0.75 | 1.69 |
| **TRBV16** | 0.02 | 0.09 |
| **TRBV18** | 1.49 | 0.99 |
| **TRBV19** | 1.73 | 2.08 |
| **TRBV20-1** | 6.67 | 6.39 |
| **TRBV21-1** | 1.03 | 1.03 |
| **TRBV23-1** | 0.72 | 0.82 |
| **TRBV24-1** | 0.90 | 1.61 |
| **TRBV25-1** | 0.48 | 1.44 |
| **TRBV27** | 4.63 | 3.59 |
| **TRBV28** | 8.20 | 3.69 |
| **TRBV29-1** | 3.90 | 6.27 |
| **TRBV30** | 0.60 | 0.80 |

**Supplementary Table 2: The frequency of variable alpha and variable beta segments in dominant and weak TCRs.** Transduced T cells expressing weak endogenous TCRs or dominant endogenous TCRs were sort-purified by flow cytometry. The endogenous TCRs were then sequenced using next generation sequencing. Shown is the percentage of each alpha variable (Table 2a) or beta variable (Table 2b) domain usage in the dominant or weak TCR populations.
